# A novel function for globulin in sequestering plant hormone: Crystal structure of *Wrightia tinctoria* 11S globulin in complex with auxin

**DOI:** 10.1038/s41598-017-04518-7

**Published:** 2017-07-05

**Authors:** Pramod Kumar, Pooja Kesari, Sonali Dhindwal, Ashish K. Choudhary, Madhusudhanarao Katiki, Neetu, Aparna Verma, Kiran Ambatipudi, Shailly Tomar, Ashwani Kumar Sharma, Girish Mishra, Pravindra Kumar

**Affiliations:** 1 Department of Biotechnology, Indian Institute of Technology Roorkee, Roorkee, Uttarakhand 247667 India; 2 Department of Botany, University of Delhi, Delhi, 110007 India

## Abstract

Auxin levels are tightly regulated within the plant cell, and its storage in the isolated cavity of proteins is a measure adopted by cells to maintain the availability of auxin. We report the first crystal structure of *Wrightia tinctoria* 11S globulin (WTG) in complex with Indole-3-acetic acid (IAA), an auxin, at 1.7 Å resolution. WTG hexamers assemble as a result of the stacking interaction between the hydrophobic surfaces of two trimers, leaving space for the binding of charged ligands. The bound auxin is stabilized by non-covalent interactions, contributed by four chains in each cavity. The presence of bound ligand was confirmed by matrix-assisted laser desorption/ionization mass spectrometry (MALDI-MS) and high-resolution mass spectrometry (HRMS). Here, we hypothesize that the cleavage of globulins by endopeptidases leads to the movement of the hydrophilic loop region from the surface to the periphery, leaving space for the binding of auxin, and promotes hexamer formation. As the process of germination proceeds, there is a change in the pH, which induces the dissociation of the hexamer and the release of auxin. The compact hexameric assembly ensures the long-term, stable storage of the hormone. This suggests a role for globulin as a novel player in auxin homeostasis.

## Introduction

Auxin is a phytohormone responsible for the regulation of tropic responses, cell division and elongation, root and shoot differentiation, development of the vascular system, abscission, senescence, and flowering in plants1. In addition, this hormone acts as a messenger for abiotic and biotic stimuli^1^. Indole-3-acetic acid (IAA) is the most abundant form of this phytohormone^2^. Gordon *et al*. showed the association of auxin with globulin and proteose seed proteins^3^. The level of free auxin in tissues is regulated *via* three basic mechanisms: synthesis, inactivation, and transportation^4^. IAA exists in a free state (active form) or in conjugated form with ester or amide forms, peptides, and proteins^5^. The auxin ester conjugates are the major inactive form of auxin found in seeds and storage organs of monocots, whereas amide conjugates are found in dicots^6, 7^. Auxin conjugated proteins have been identified in seeds of *Phaseolus vulgaris*, *Arabidopsis thaliana*, *Pisum sativum* and *Fragaria ananassa* ^8^. The conjugation of auxin and protein depends on the particular protein, as well as on the particular plant species^9^.

Plant seeds are a reservoir of proteins required during the different phases of seed development and survival. For example, lectin, Kunitz trypsin inhibitor, albumin, and chitinase are involved in a defensive role^10–15^, whereas the nutritional requirement of the seed is defined by globulins, which represent the most widely distributed group of seed-storage protein^16^. These storage proteins are synthesized during seed formation, accumulate in the endosperm layer and are used in later stages of seed development. Globulins are a class of storage proteins that belong to the most conserved and functionally diverse cupin superfamily^17, 18^. The polypeptide precursor of globulin (pre-pro-globulin) is synthesized in the rough endoplasmic reticulum (ER)^19^. The N-terminal signal sequence directs the precursor towards the lumen of the ER where it is co-translationally cleaved and form pro-globulin. ATP helps in the efficient assembly of pro-globulin trimers, which are sorted to the protein storage vacuole (PSV) by vesicles^20^. The pro-globulin consists of an acidic and a basic subunit bridged by a disulphide bond^21^. In the PSV, post-translational cleavage of the bond between two cupin domain at the conserved Asn-Gly site by the vacuolar processing enzymes (VPE) asparaginyl endopeptidase results in a conformational change in the monomers of the trimers, ultimately resulting in the formation of a hexamer^22, 23^. In its hexameric state, all Asn residues become shielded and resistant to further proteolysis by the processing enzyme. If the pro-globulin is incorrectly folded and the cleavage takes place at another Asn residue, the VPE continues to digest the storage protein^24, 25^. Thus, processing of the 11S globulin increases this protein’s ability to form stable protein bodies, to withstand desiccation, and to be rapidly hydrolyzed by the proteolytic enzymes of the germinating seed. The change in pH of the storage vacuoles activates the proteases^26^ and initiates the selective breakdown of globulin to release nutrients and energy for seed germination and early seedling growth^27, 28^. The acidic domain undergoes proteolytic cleavage while it still remains bonded to the basic chains. 11S globulins predominantly exist as hexamers due to the non-covalent interactions between the trimers^29^. Seed globulin has been shown to bind charged ligands in its hydrophobic cavity^30^.

In this study, we report the crystal structure of 11S homo-hexameric globulin complexed with Indole-3-acetic acid (*IAA*), an auxin, isolated from seeds of *Wrightia tinctoria* at 1.7 Å resolution. To the best of our knowledge, we are presenting the first structure of the complex of auxin sandwiched between two trimers of 11S globulin. Three hydrophobic pockets coordinating auxin molecule show symmetrical hydrogen-bonding pattern between the ligand and the protein. The matrix-assisted laser desorption/ionization mass spectrometry (MALDI-MS) and high-resolution mass spectrometry (HRMS) confirmed the presence of auxin in *Wrightia tinctoria* globulin (WTG). Auxin being an essential hormone is utilized in most of the growth and development processes of plants. Results demonstrate a novel function of WTG in the storage of plant hormones that are essential for seed germination apart from its nutritional importance in seeds.

## Results and Discussion

### WTG structure determination and refinement

The crystal structure of WTG was determined at a final resolution of 1.7 Å. It belongs to *P*2_1_2_1_2_1_ space group and has six monomers in an asymmetric unit forming two trimers. The *R*_factor_ and *R*_free_ values are 17.4 and 22.7, respectively. The final statistics of data collection and refinement parameters are described in Table 1. The Ramachandran plot for analysis of the main chain conformation showed that 96% residues are lying in favoured regions, while two residues (Glu461 of chain D and Gln206 of chain E) are present in the disallowed region. The residues Glu461 lie at the C-terminus of the protein. The structural superposition of chain A to the other five chains shows an average Cα RMSD of 0.11 Å confirming that all the monomers of WTG are essentially identical. The density for the first 26 N-terminal residues (signal peptide sequence) was not observed because of the post-translational cleavage of the signal peptide. The electron density corresponding to 18 residues of the C-terminus region was also absent. In addition, the structure has auxin at the three interfaces of four chains as distinctly shown by difference Fourier maps. Densities were also observed for two phosphates (PO4), one citrate (FLC) (both contributed by the crystallization buffer), and glycerol (GOL) (used as cryoprotectant).

**Table 1:** Summary of data collection, structure refinement, and validation statistics.

PDB ID	5WXU
Beamline	ESRF BM14 Grenoble
Crystallographic data	
Resolution (Å)	101–1.7 (1.73–1.70)
Space group	*P* 2_1_ 2_1_ 2_1_
Cell dimensions	
*a, b, c* (Å)	111.21, 114.25, 202.47
Total reflections	1067915
Unique reflections	270464
Multiplicity (Last shell)	3.9 (3.3)
Completeness (%) (Last shell)	96.05 (92.87)
CC1/2 outer shell	0.826
Mean I/σ (Last shell)	16.77 (1.97)
Wilson B-factor	21.42
Matthews coefficient	2.40
R_sym_ (%)a (Last Shell)	0.063 (0.826)
Refinement	
*R*_factor_ (%)	17.4
*R*_free_ (%)	22.7
Number of atoms	21962
Macromolecules	19550
Ligands	351
Water molecules	2091
Protein residues	2388
RMSD on bond lengths (Å)	0.021
RMSD on bond angles (Å)	2.21
Average B-factor (Å2)	27.90
Macromolecules	26.30
Water	40.20
Ramachandran favoured (%)	96
Ramachandran outliers (%)	0.17
Clashscore	10.92

*Statistics for the highest-resolution shell are shown in parentheses.

^a^R_sym_ = Σ_hkl_ Σ_i_^n^_=1_|I_hkl,i_ − [overbar]I_hkl_|/Σ_hkl_Σ_i_^n^_=1_|I_hkl,i_.

### Overall structure of WTG

**Figure 1:**
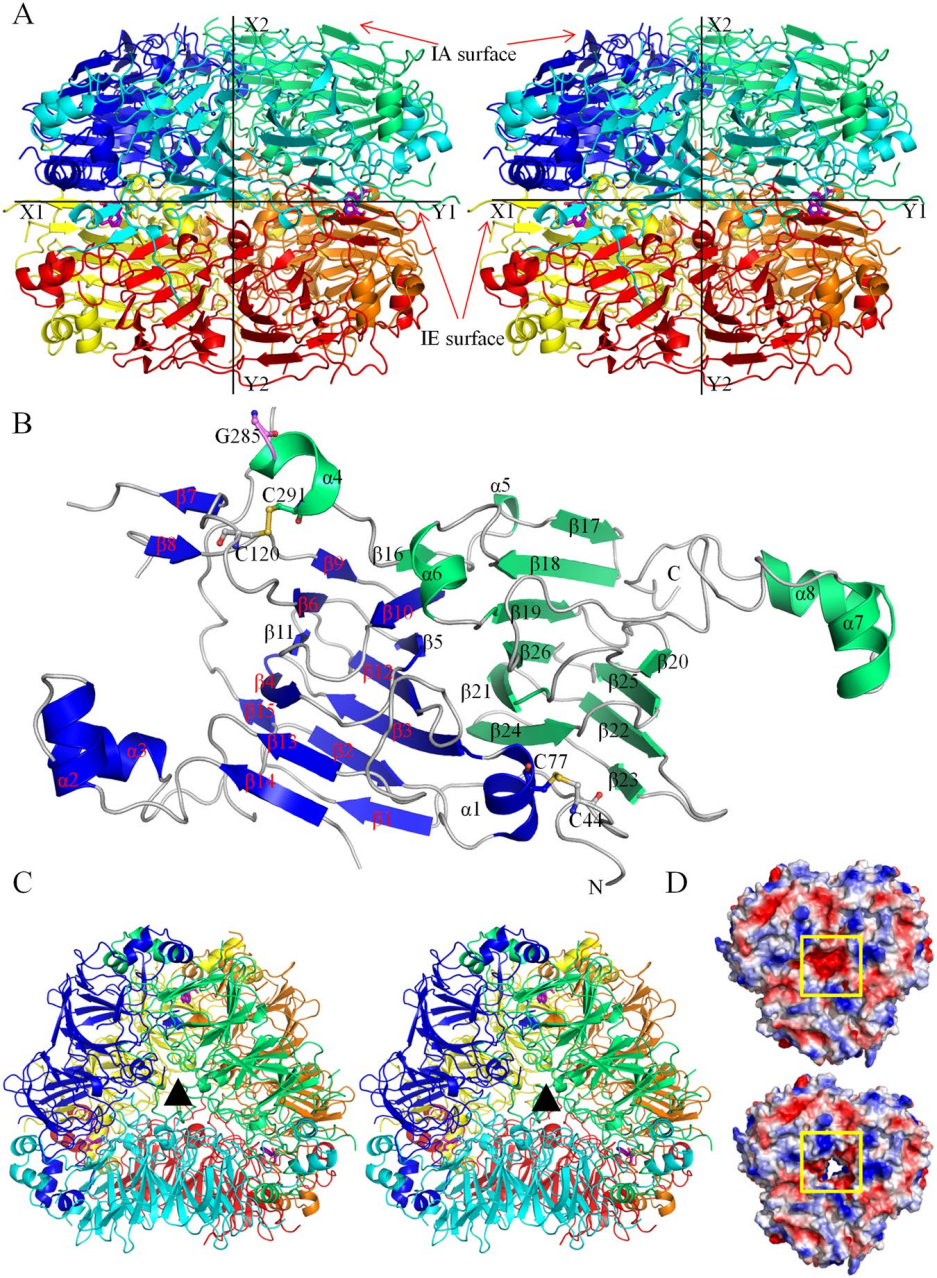
The overall architecture of 11S globulin from *Wrightia tinctoria*. (**A**) The stereoview of the two-fold axis of symmetry of WTG hexamer. The X1-Y1 axis represents the two-fold axis of symmetry between the two trimers of WTG. The X2-Y2 axis represents the threefold axis of symmetry present in the trimers of WTG. The IA and IE surfaces of the trimers have been indicated by red arrows. All the chains of the WTG hexamer are represented in cartoon form. Six monomers are shown in blue (chain A), green (chain B), cyan (chain C), yellow (chain D), red (chain E), and orange (chain F). (**B**) The WTG monomer depicting acidic subunit (blue) and basic subunit (green) having 26 β-sheets and 8 α-helices. The Gly285 asparaginyl endopeptidase cleavage site, intra-subunit (Cys44-Cys77) and inter-subunit (Cys120-Cys291) disulphide bonds are represented as ball-and-stick. The extended alpha helix and bicupin fold have been indicated. (**C**) The stereoview of the three-fold axis of symmetry of WTG trimer from the top. The triangle represents the axis perpendicular to the three-fold axis of symmetry. (**D**) The electrostatic surface potential of intra-subunit (IA) surface of trimer ABC in the presence and absence of Ser27-Ser34 residues, respectively. The region covers the hydrophilic channel formed at the center of the trimer (yellow box).

An asymmetric unit consists of six monomers arranged in hexameric assembly (Fig. 1). These six monomers of WTG can be divided into two trimers (chains ABC and DEF); that are stacked together through hydrophobic and hydrogen-bonding interactions. In the structure, the N-terminus of chain A is stacked over the C-terminus of chain D, the N-terminus of chain B is over the C-terminus of chain F and the N-terminus of chain C is over the C-terminus of chain E. The sandwiched face of the trimer is designated as inter-chain disulphide containing (IE), whereas the outer face is referred to as intra-chain disulphide-containing (IA) on the basis of the inter- and intra-chain disulphide bonds position, respectively^28^. An axis through the sandwiched face of the trimer shows that the hexamer exhibits two-fold symmetry (Fig. 1A).

### WTG Monomer

The WTG monomer consists of 8 α-helices and 26 β-strands that can be divided into two domains: the N-terminal domain and the C-terminal domain (Fig. 1B). A pseudo-dyad symmetry was found in the monomers. The overall architecture of a WTG monomer is similar to the previously reported 11S globulin from soybean^28^. Both domains of WTG contain a similar structural topology but differ in length (284 and 195 residues), as well as in iso-electric point (6.16 and 8.59, respectively). This observation clearly indicates that the N-terminal domain is acidic and the C-terminal domain is basic. The core region of both the N- and C-terminal domains contains the β-barrel cupin fold extended by helices on both sides. The α-helices H2, H3 of the acidic subunit and H7, H8 of the basic subunit constitute the extended α-helical signature domain of the seed storage globulins. The acidic and the basic domains are joined together by a disulphide bond (Cys120-Cys291), two salt bridges (Glu53-Arg381 and Asp158-Lys296) and hydrogen bonds. Additionally, an intra-chain disulphide bond between the Cys44 of the flexible N-terminal loop and Cys77 of the first α-helix helps stabilize the N-terminal loop region of the acidic domain. The inter-chain disulphide (IE) bond between acidic and basic subunit is well conserved among all the 11S class globulins^23^.

The 11S globulins contain four disorders region of variable length. No density was observed for any of these four disordered regions (residues: 27–39, 131–142, 208–218, and 274–284) except for a few residues (Ser27- Ser34) of the loop region I. The disordered loop region I is localized near the centre of the trimer and covers the hydrophilic channel formed at the centre of the trimer along the threefold axis making molecules inaccessible to the IE surface (Fig. 1D). The disordered region III is localized on the IA surface of the trimer, whereas the disorder region II and IV are localized on the IE surface of the trimer. The disorder region IV contains the cleavage site (Asn284-Gly285 in WTG) for asparaginyl endopeptidase. The cleavage at Asn284-Gly285 releases the disorder region IV (residue 274–284) and initiates the interaction between the IE faces of the trimer.

### Interactions stabilizing the WTG trimer

The trimer possesses a three-fold symmetry (Fig. 1C). About 15% of the available surface area of a monomer is buried upon interaction with the adjacent monomer. The acidic subunit of one monomer interacts with the basic subunit of another monomer through their α-helical domains, helix-barrel domains, and barrel domains. A high degree of hydrophobic interactions and hydrogen-bond formation stabilizes the trimerization. The salt bridges are formed among Arg28, Glu122, Asp146, Asp230, Lys247, Arg266, and Lys372 of one monomer and Asp185, Arg427, Lys449, Arg364, Glu444, Asp432, and Glu251 of another monomer, respectively.

### Interaction between IE surfaces

**Figure 2:**
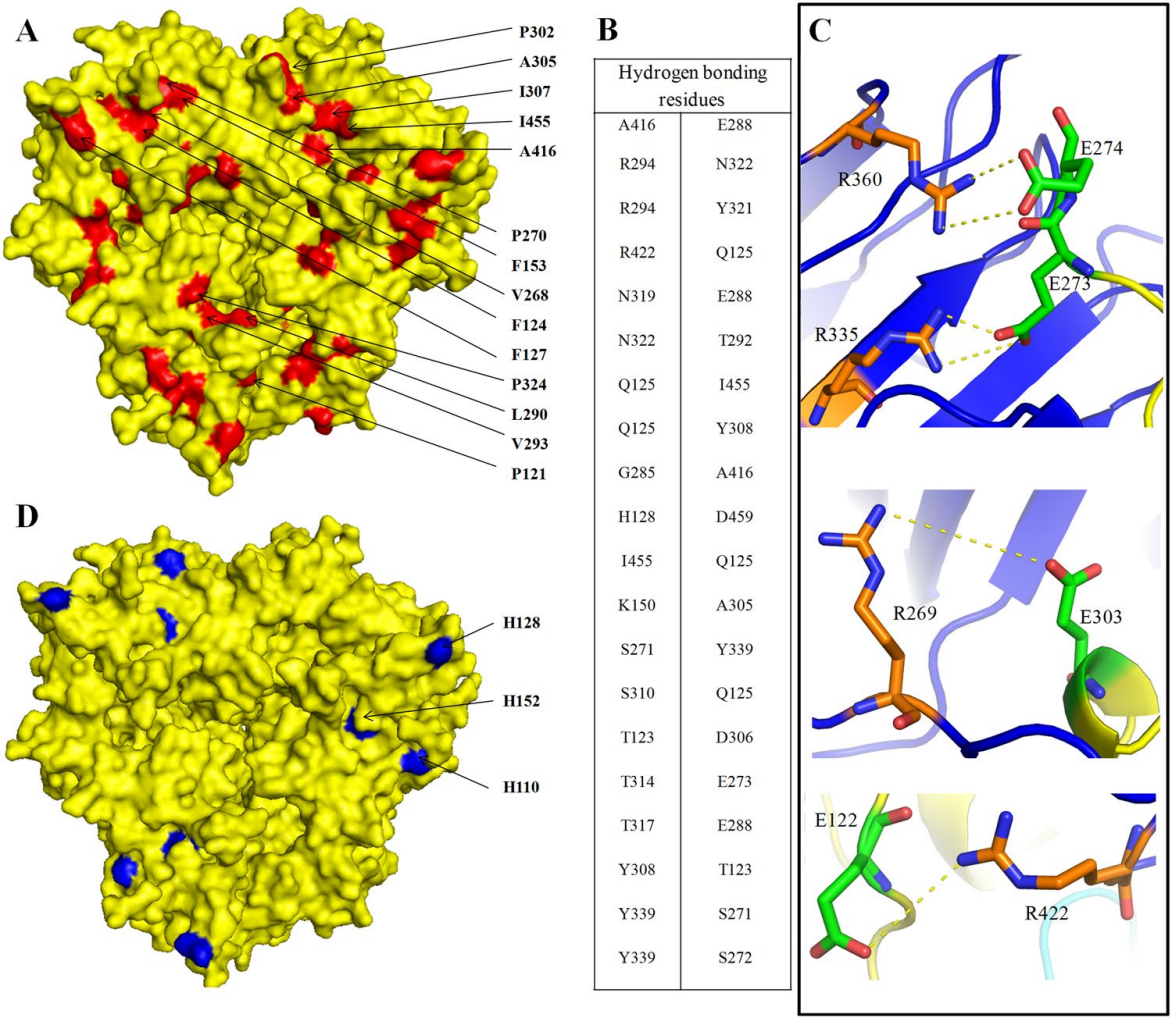
Protein-protein interaction involved in the formation of WTG hexamer. (**A**) The surface view of WTG trimer (yellow) showing the hydrophobic residues (red) involved in interaction with residues of another trimer. (**B**) The residue pairs of chain A and D involved in hydrogen bond formation. The N-terminal of chain A is stacked over C-terminal of chain D during hexamer formation. (**C**) The key residues involved in salt bridge formation between the trimers. Residues from chain A (orange sticks) and residues from chain D (green sticks). The interactions are shown via yellow dashed lines. (**D**) Residues His110, His128 and His152 present on the surface become more positively charged at acidic pH and result in dissociation of IE face. All residues have been shown in ball and stick representation.

Disordered region IV is composed of hydrophilic residues that hinder the stacking of the trimer. In the PSV, the cleavage of the Asn284-Gly285 bond releases the loop region from one end reducing the steric hindrance and tight packing of the trimers. The trimers stack together to form a hexamer for efficient space utilization and long term storage in the PSV. The individual accessible surface area (ASA) of the ABC and DEF trimers are 42,327.6 Å^2^ and 42,475.8 Å^2^, respectively; however, the interaction between IE surfaces in the hexamer reduces the ASA to 63119.5 Å^2^. Electrostatic surface analysis of the IE surface revealed the presence of charged amino acids and hydrophobic amino acid; favours hydrophobic interactions (Fig. 2A), hydrogen bonds (Fig. 2B), van der Waals contacts, and salt bridges between the two trimer surfaces (Fig. 2C). Interestingly, most of these residues are highly conserved among the members of the 11S family. The salt bridges are formed in and around disordered loop region IV (Fig. 2C). Most of the charged residues *i.e*. Arg155, Asp306, and Arg422 present on the IE surface are well conserved in all the reported structures of 11S globulin.

### Ligands bound to IE surface

The electron-density map at 1.7 Å shows that the IE surface of the trimer binds many ligands (citrate, phosphate, PEG, and glycerol) from the crystallization buffer and cryoprotectant. Surprisingly, a clear electron density was also observed for a naturally bound auxin-like molecule, and we observed three symmetrical pockets with similar electron densities for such a ligand.

In the crystal structure, the phosphate and citrate anions are localized to the IE surface (Supplementary Figure S1A). Citrate and phosphate bind to the positively charged region on the IE surface of both trimers. In all the chains, citrate (Supplementary Figure S1B) interacts with backbone amino groups of Phe153, Ile295, Lys296, and the side-chain amine group of Arg294 through its carboxylic moiety. One phosphate interacts with residues His85, Ser96, Asn98, His168, His184 and Arg199 (Supplementary Figure S1D) within each monomer, whereas the other phosphate lies at the interface of two monomers such that it interacts with residues Lys415 and Tyr321 of one monomer, and Thr289 from another monomer (Supplementary Figure S1C).

### Putative auxin binding pocket

**Figure 3:**
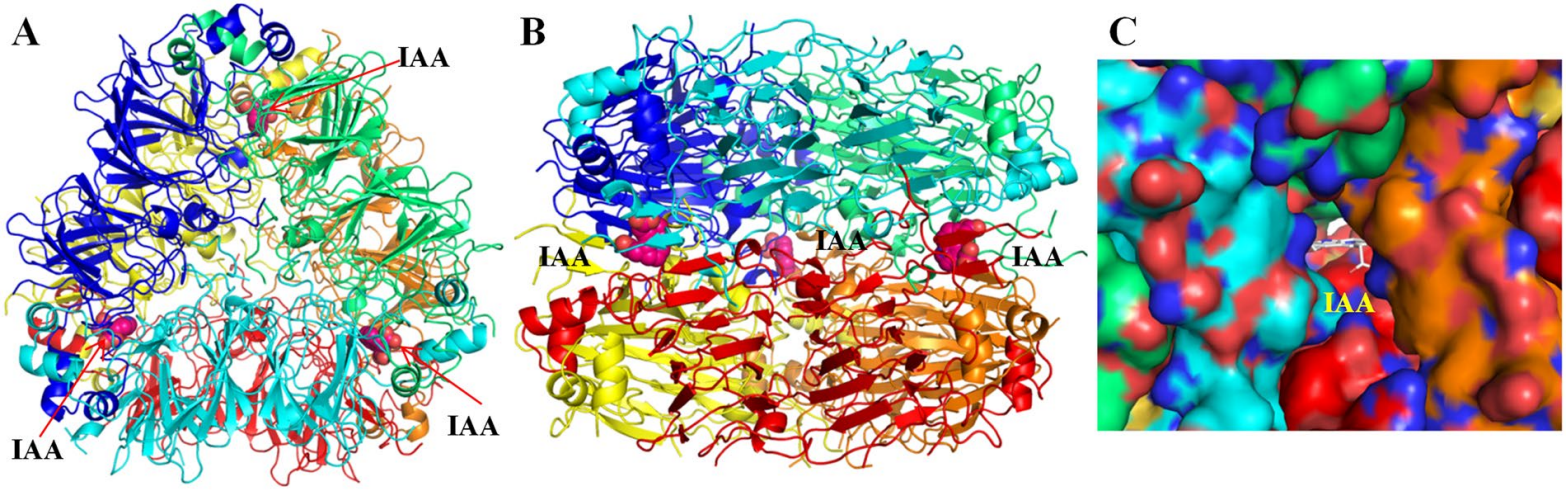
The auxin-binding site architecture. (**A**) The WTG hexamer showing three symmetrical auxin binding pockets each formed by four WTG monomers. (**B**) The side view of WTG hexamer showing auxin binding pockets sandwiched between two trimers. The auxin is shown as pink spheres whereas WTG hexamer is shown in cartoon representation. All the chains have been shown in different colours - chain A (blue), chain B (green), chain C (cyan), chain D (yellow), chain E (red), and chain F (orange). (**C**) Surface view of WTG tetramer demonstrating the size and shape of the auxin-binding pocket with auxin shown in white sticks. All the C-alpha atoms of different chains have been shown in different colors - chain A (blue), chain B (green), chain E (dark red), and chain F (orange) in surface view. The light red and dark blue color show surface of oxygen and nitrogen in all the four chains.

**Figure 4:**
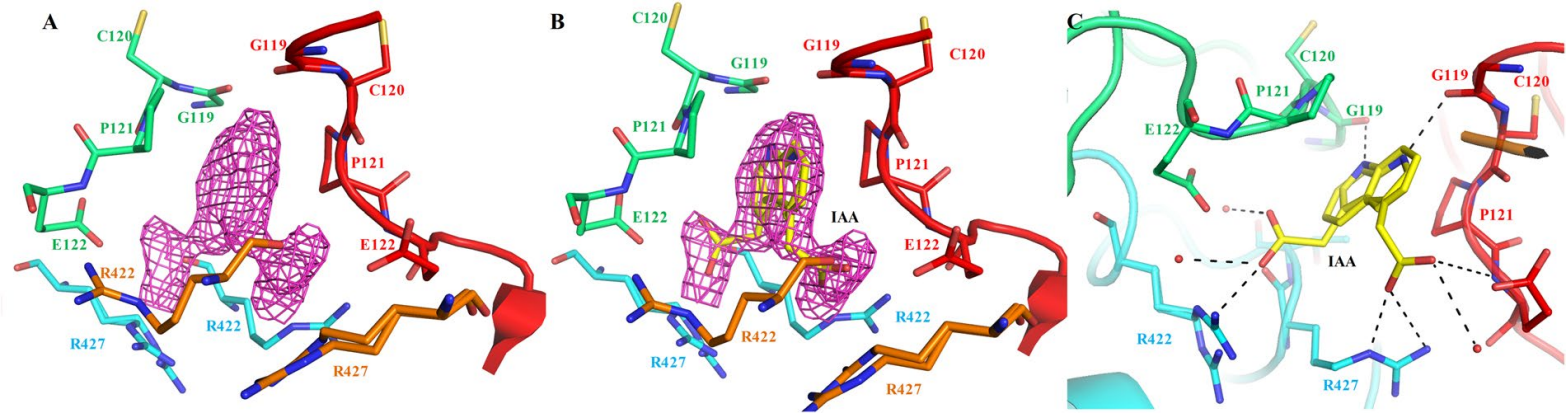
The structural insights of putative auxin binding site. (**A**) Close-up view of ligand binding pocket showing Fobs-Fcalc electron density map contoured at 2.5σ. (**B**) Auxin molecule show in two different orientation (shown as sticks) fitted in the *F*_obs_-*F*_calc_ electron density map. (**C**) Intermolecular hydrogen bonds between the auxin molecule and binding site residues formed by chains B, C, E and F. WTG hexamer is shown in cartoon representation with chain B (green), chain C (cyan), chain E (red), and chain F (orange). Auxin molecule and side chains of residues are shown as sticks. The water molecules are represented as spheres. The hydrogen bonds are shown as dashed black lines.

Previous reports show that auxin binds to the hydrophobic pockets of auxin-binding protein ABP1, through its indole ring^31^. ABP1 is a monocupin protein in which the binding site for auxin lies inside the barrel domain (~11 Å inside from the protein surface). In ABP1, the IAA in the cavity is stabilized by Zn ion, and few H-bonds. Interestingly, the size and shape of the density present in WTG at 1.7 Å resolution structure suggested that it can accommodate an auxin-like molecule. The hydrophobic pocket formed at the interface of the tetramer (adjacent dimer from each trimer) on the IE surface sandwiched between two trimers act as the site for binding of the ligand (Fig. 3A–C). Since the precise nature of this naturally bound ligand was unknown, we utilized the structure of indole acetic acid for the refinement process. Inspection of electron-density maps (2*F*_o_-*F*_c_ at 1σ and *F*_o_-*F*_c_ at 2.5σ) showed clear density for three auxin-like molecules. The *F*_o_-*F*_c_ map confirmed the existence of the ligand in the electron density (Fig. 4A and B). The indole acetic acid matched the electron density and its *B*-factor was similar to those of the protein residues. There was no density for any metal ion in all the three pockets. Also, no metal coordinating residues were observed in the auxin binding pockets.

The precise packing of the molecule in the cavity shows that it interacts well with the residues lining the pocket. The indole ring snuggles into the cavity formed by proline residues of the acidic subunit of two monomers from opposite trimers, whereas the carboxyl group interacts with residues contributed by the basic subunit of two other monomers. All three pockets are related by a three-fold axis of symmetry formed by residues Gly119-Glu122 of chains A and F, and residues 423–427 of chains B and D; Gly119-Glu122 of chains B and E, and 423–427 of chains C and F; and Gly119-Glu122 of chains C and D and 423–427 of chains A and E. The residue patch formed by Gly119-Glu122 is part of a long flexible loop region that involves the highly conserved Cys120-Cys291 disulphide bond. Residues Arg422 and Arg427 are contributed by the loop region connecting the β-barrel domain to the extended α-helical region of the C-terminus. The pocket lies in close proximity to the asparaginyl-endopeptidase cleavage site.

### Interaction with auxin

Indole-3-acetic acid (IAA) is the main form of auxin found in the plants^2^. As discussed previously, the structure of WTG contains an electron density that accommodated an auxin-like compound. The 2*F*_o_-*F*_c_ Fourier map at 1σ clearly revealed the presence of an IAA molecule in alternate orientation at the hydrophobic site formed by chain BCEF (Fig. 4A and B). Therefore, an alternate orientation of IAA (0.5 occupancy) was positioned in the site and it was entrenched in the electron-density map. IAA binds to the pocket through two of its important functional moieties, the indole ring, and the carboxyl side chain. The indole ring goes deep in the pocket and makes pi-sigma interaction with Pro121 of two chains lying diagonally opposite to each other. The two orientations of auxin shares similar interactions because the residues involved from chain B and C are symmetrically related to the corresponding residues from chain E and F, respectively (Fig. 4C). In one orientation, the amine group of the indole ring bonds with the backbone oxygen atom of Gly119 of chain E. The carboxyl side chain oxygen atom of auxin interacts with side chain NH_2_ and NE atoms of Arg427 of chain C and water molecules in the nearby vicinity. Another carboxyl side-chain atom of auxin interacts with side chain OE1 atom of Glu122 of chain E and the backbone oxygen atom of Arg422 of chain C.

In the second orientation, the amine group of the indole ring bonds with the backbone oxygen atom of Gly119 of chain B. The carboxyl side-chain atom of auxin interacts with the NH backbone and OE1 side chain atoms of Glu122 of chain A. The other carboxyl side-chain atom of auxin interacts with side chain OE1 atom of Glu122 of chain A and the backbone oxygen atom of Arg422 of chain D. In the cavity formed at the interface of other chains of the hexamer, the carboxyl side-chain atom of auxin sometimes shifts more towards chain E and interacts with a side-chain OE2 atom of Glu122 and the backbone oxygen atom of Ser424 through water bridges.

The direct and indirect (through water bridges) hydrogen-bonding networks conferred by four chains precisely balance the alternate orientation of auxin. The interaction pattern was found to be same in all the symmetrical pockets. Along with auxin, there was a clear density for a PEG ion. PEG interacts with auxin and stabilizes it inside the hydrophobic cavity.

### Confirmation of ligand by matrix-assisted laser desorption/ionization mass spectrometry (MALDI-MS) and high-resolution mass spectrometry (HRMS)

**Figure 5:**
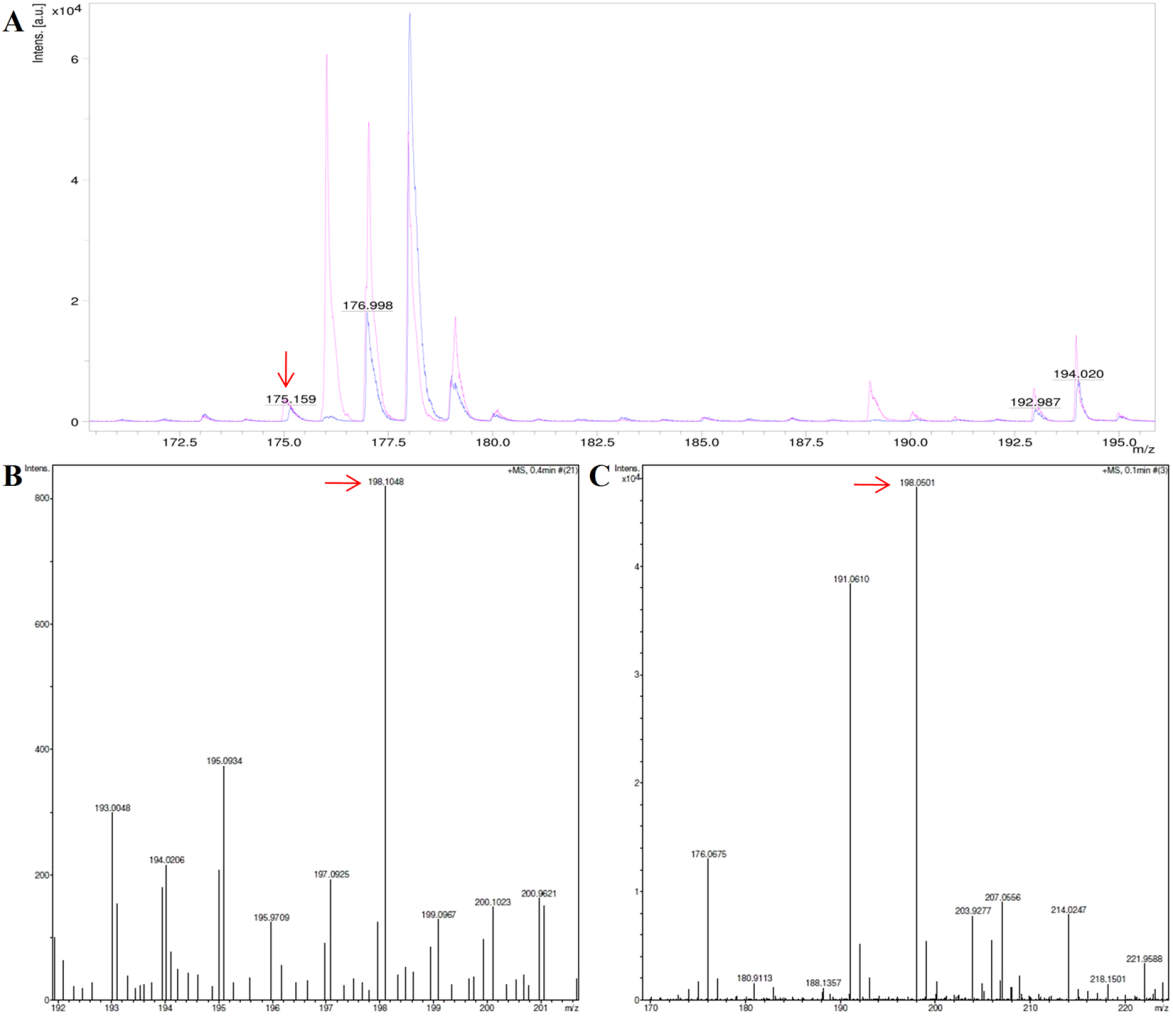
Ligand identification of by MALDI-MS and HRMS. (**A**) MALDI-MS spectra profile of pure Indole-3-acetic acid (IAA) acquired from Sigma-Aldrich (magenta) and IAA obtained from denatured WTG sample (blue). The auxin (IAA) corresponds to a molecule with m/z = 175.15 (shown by red arrow). In addition, the spectrum also showed peaks in the low molecular weight range from 100–500 Da indicating peaks corresponding to the matrix ions. (**B**) HRMS profile of sample isolated from purified WTG wild-type protein. (**C**) HRMS profile of standard auxin. A peak at 198.10 corresponding to an m/z ratio of the IAA sodium adduct C_10_H_9_NO_2_Na [M + Na]^+^ was obtained. The red arrow in B and C indicate the m/z ratio of the IAA sodium adduct.

To investigate the presence of auxin with the wild type WTG protein, as suggested by the crystallographic data, initially MALDI-MS was performed to confirm the average molecular weight. To this end, the ligand sample was isolated from the purified WTG protein and pure indole-3-acetic acid (IAA) (procured from Sigma-Aldrich, USA) was used as a reference. Standard IAA was calibrated with an m/z peak corresponding to [M + H]^+^ at 175.159, while the average molecular weight of auxin was confirmed by the overlap of MS peaks with the standard as shown Fig. 5A. Of note, background peaks were observed (Supplementary Figure S2), arising from the matrix cluster formation due to the usage of conventional matrices (e.g. DHB) in the detection of small molecules (100–1000 Da)^32^.

To further validate the presence of IAA, the lyophilized auxin sample was analyzed by HRMS using Brüker micrOTOFTM-Q II mass spectrometer. For analysis, the 20 µL of auxin sample was dissolved in acetonitrile and injected into the MS. A peak was detected in HRMS at m/z 198.10 corresponding to IAA with sodium adduct C_10_H_9_NO_2_Na [M + Na]^+^. Figure 5B shows the HRMS spectrum of auxin sample isolated from purified WTG wild-type protein, while Fig. 5C shows the spectrum of standard auxin. The results obtained from MALDI-MS and HRMS confirm the molecule associated with WTG is, indeed, indole −3 acetic acid.

### Sequence and structural comparisons with other 11S Globulin

**Figure 6:**
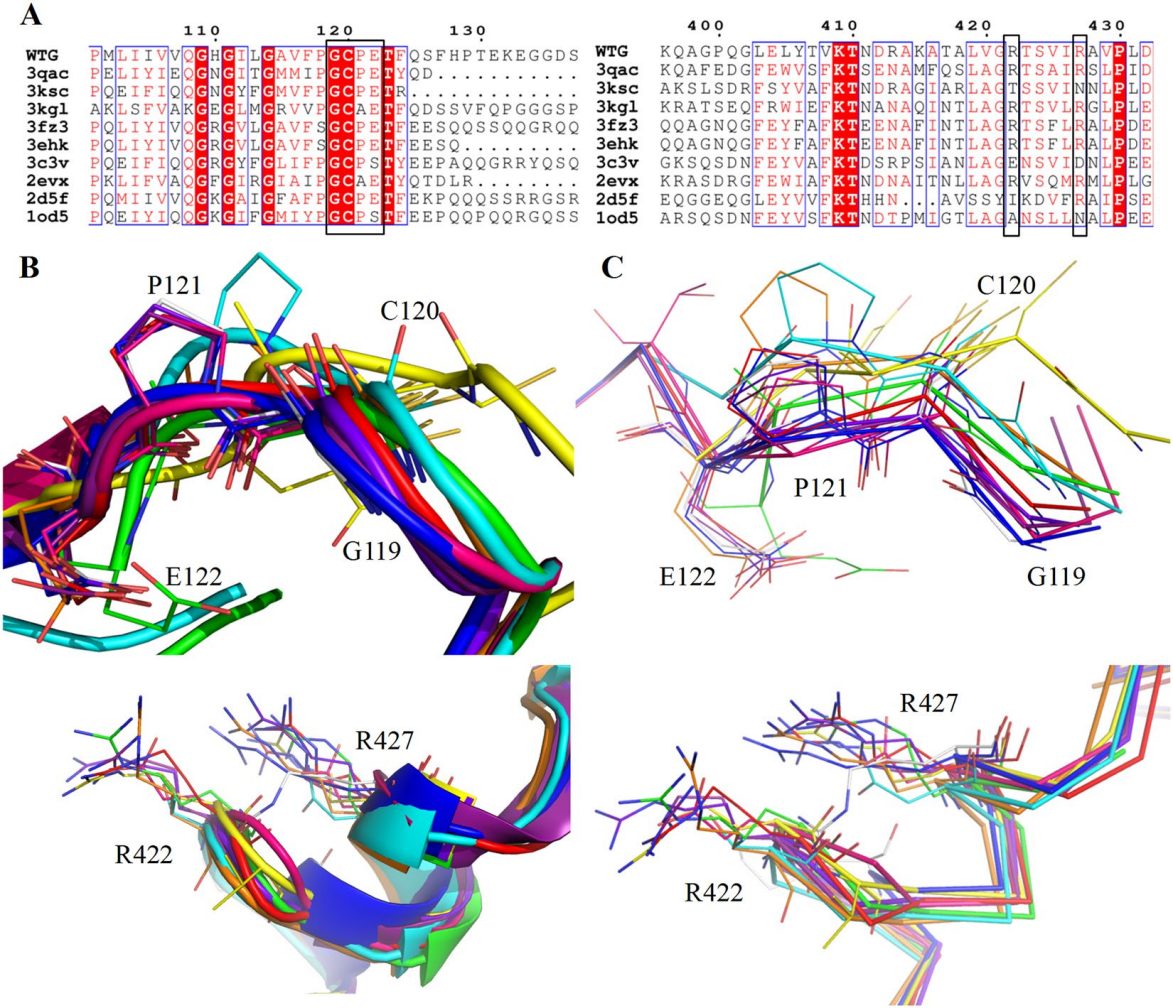
Sequence and structural characteristics of auxin-binding site residues. (**A**) Sequence alignment of auxin-binding site residues of WTG with other related members of the 11S family. Plausible auxin binding residues are enclosed in a black box. Identical residues are shown as red boxes with white characters; red characters denote identical residues across the column, whereas blue boxes denote similar residues. The sequence alignment was performed using ClustalW and the figure generated using ESPript2. (**B**) Structural superimpositions of the auxin-binding site residues present in WTG and corresponding residues from the previously determined 11S globulin structures reveal the presence of identical residues. (**C**) The key residues involved in auxin binding described in WTG (Gly119, Cys120, Pro121, Glu122, Arg422 and Arg427) and corresponding residues of other 11S globulins are represented as sticks. WTG (red), 3qac (orange), 2evx (yellow), 3kgl (green), 2d5f (blue), 1od5 (white), 3ehk (purple), 3c3v (pink), 3ksc (cyan), and 3fz3 (violet).

Members of the 11S globulin superfamily share overall low sequence homology but high structural homology. A sequence alignment of 11S globulin with available crystal structures and WTG revealed significant conservation in auxin binding residues (Fig. 6A). The alignment of the full-length sequences of known 11S globulin crystal structures with a sequence of WTG is shown in Supplementary Figure S3. In particular, the region containing residues Gly119-Glu122 is well-conserved. Gly119 is involved in anchoring the indole ring of auxin to the cavity, whereas the Cys120 is the part of the inter-chain disulphide bond; the Cys120-Cys291 disulphide bond is a well-conserved characteristic of the 11S family. The hydrophobic residue Pro121, replaced by Ala in certain 11S globulins, also makes the cavity hydrophobic. In some members of the 11S family, a Ser residue exists in place of Glu. We hypothesize that the mutation of Glu122Ser will allow the carboxyl group of auxin to interact with surrounding water molecules and stabilize itself in the vicinity. The Arg422 and Arg427 residues that support the carboxyl end of the auxin exhibit moderate conservation. In all the 11S structures, these positions are mostly occupied by charged residues Lys or Arg.

The structural comparisons through DALI search engine showed that prunin (PDB ID: 3fz3), Pru Du Amandin (PDB ID: 3ehk), glycinin A3B4 subunit (PDB ID: 2d5f), arachin ARAH3 (PDB ID: 3c3v), glycinin (PDB ID: 1od5), Prolegumin (PDB ID: 3ksc), Amaranthus (PDB ID: 3qac), 11S proglobulin pumpkin (PDB ID: 2e9q), Procruciferin from *Brassica napus* (PDB ID: 3kgl), 11S globulin pumpkin (PDB ID: 2evx), glycinin G1 (PDB ID: 1fxz), and cocosin (PDB ID: 1xgf) share high structural homology (Cα RMSD less than 2 Å) with WTG. Supplementary Figure S4 shows the Cα RMSD plot per residues of these globulin sequences.

Most of these 11S globulin structures bind to anionic ligands like (such as, carbonate, chloride, and sulphate) and metal cations (Ca^2+^, Mg^2+^, and Na^+^). These ligands help stabilize the globulin structure. The structure of glycinin A3B4 subunit (PDB ID: 2d5f) and glycinin (PDB ID: 1od5) have binding sites for carbonate and Mg^2+^ ion inside the barrel domain. Carbonate coordinates with Arg50, His58, Tyr131, and Thr133, whereas Mg^2+^ coordinates with His383, Glu432, and Tyr441. In 11S globulin from pumpkin (PDB ID: 2evx), Mg^2+^ ion coordinates with Lys387 and Arg447. No electron-density was observed for any metal cations in the structure of WTG. The structure of prunin (PDB ID: 3fz3), contains Ca^2+^ and Na^+^ ion bound on the IE surface of the trimer (Supplementary Figure S5). Superimposition of the hexamer assembly of prunin over that of WTG showed that the binding site for Ca^2+^ in all six chains of prunin was occupied by citrate ion in all six chains of WTG. Moreover, the three Na^+^ ion-binding sites lying on the IE surface of prunin are close to the auxin-binding site of WTG; however, no density for any metal ion was observed at this site in WTG. Sequence and structural analysis reveal that residues involved in stabilization of the auxin molecules in the hydrophobic pocket were mostly conserved among all the reported 11S globulin structures (Fig. 6B and C). However, slight variations in the pocket residues may help globulin in the storage of different forms of metabolic ligands.

### Biological Implications

The plant hormone auxin is stored in different tissues in a variety of forms. Plant vacuoles are the highly specialized and dedicated compartments that store many plant metabolites like hormones. Vacuoles play a key role in hormone signaling and homeostasis, by facilitating their storage and regulating their time-dependent release for their corresponding roles during plant growth^33, 34^. The vacuoles also store seed storage proteins, 11S globulins, which are the nutrient reservoir of the seed. The post-translational processing of pro-globulin trimers by asparaginyl endopeptidase exposes the hydrophobic IE surface to solvent and triggers hexamer formation for its stable long-term storage^22, 24^. The IE surfaces are rich in hydrophobic residues and acts as sites that accommodate metabolic ligands.

Our structural analysis showed that metabolic ligands (i.e. auxin) bind to the hydrophobic cavity lined by the Cys120-Cys291 disulphide on the IE surface. The residues surrounding the auxin-binding pocket support the tight binding of the molecule through hydrophobic and hydrogen-bond interactions. The hydrophobic IE surfaces of the two trimer stack together to prevent further proteolysis^25, 35^. Since the endopeptidase cleavage site (Gly285) is near (~5 Å) Pro121 of another chain, it can be assumed that the binding of an auxin molecule and hexamer formation are simultaneous events and, apparently, take place during the stacking of two trimers. During this phase, the auxin buries itself in the cavity, and the trimers interact to form a hexamer.

Electrostatic attraction-repulsion is the driving force for protein-protein interactions. As germination proceeds, the PSV becomes more acidic^36^, which transfer positive charge on the histidine residues of the IE face (Supplementary Figure S1D). This distribution of potential charges on the IE surface favours dissociation due to the repulsion between the two faces^28^. Repulsion will open the hydrophobic surface on the globulin (thus, releasing auxin) and initiates the degradation of 11S globulin. This mechanism may regulate the release of auxin hormone and degradation of 11S globulins in a temporal fashion. This can be correlated with the dense packaging and long-term stable storage of such molecules inside the cavity and release during the seed germination.

## Experimental Procedures

### Preparation of WTG Crystals

Purification, crystallization and preliminary X-ray diffraction profile have been reported earlier^37^. Initial crystals of WTG were obtained in 0.2 M sodium citrate tribasic dihydrate, 100 mM Tris hydrochloride pH 8.5, 30% v/v PEG 400. 5% glycerol was used as a cryoprotectant.

### Data collection, Structure determination, and Refinement

The diffraction images were collected at synchrotron radiation source, ESRF Grenoble (BM14 beamline). The diffraction data were indexed, integrated and scaled using the HKL2000 program suite^38^. The determination of initial phases was done by molecular replacement using program MOLREP^39^ under the CCP4 software suite of version 6.0^40^. The crystal structure of Pru du amandin (PDB ID: 3ehk) was used as a search model^41^. The structure was refined by rigid-body refinement followed by iterated cycle of restrained refinement for atomic parameters by using the program REFMAC 5.8^42^. Model building was done using the program COOT^43^. The IAA molecule was refined with partial (0.5) occupancy in both orientations. The stereochemical properties of the model were evaluated using PROCHECK^44^ and MolProbity^45^. The coordinates and structure factor amplitudes were deposited to Protein Data Bank with PDB ID ‘5WXU’. PISA (Protein Interfaces, Surfaces, and Assemblies) tool was used to analyze surface area and the interactions taking place between the surfaces^46^. The electrostatic potential of the protein surfaces was calculated using PyMOL plugin “APBS”. Figures of the protein model were prepared using the program PyMOL^47^.

### Sequence alignment, pI calculation and protein-ligand interaction

The PSI-BLAST algorithm^48^ was used to identify the sequences of previously reported 11S globulin in PDB that are homologous to WTG. Sequence alignment was performed using programs CLUSTALW^49^ and ESPript^50^. The theoretical expected pI of the N-terminal and C-terminal monomers of WTG was calculated using ProtParam^51^. The protein-ligand interaction diagrams were prepared using LigPlot+^52^.

### Identification of ligand by matrix-assisted laser desorption/ionization mass spectrometry (MALDI-MS) and high-resolution mass spectrometry (HRMS)

For the confirmation of the crystallographically identified ligand, purified WTG fractions from the seeds of *Wrightia tinctoria* were concentrated using Amicon® Ultra Centrifugal Filters (10kDa-cutoff) and extensively dialyzed against Tris buffer pH 7.4 prepared in HPLC-grade water to remove any unbound molecules because WTG was isolated from a native source. After dialysis, protein was denatured using 8 M urea and precipitated using acetone followed by centrifugation at 11,000 rpm for 1 hour. The supernatant was collected and dried at room temperature in a speedy-Vac (CentriVap Centrifugal Vacuum Concentrators). The sample was divided into two aliquots, with one analyzed by MALDI-MS and other by HRMS. For MALDI-MS, auxin sample (isolated from WTG fractions) and standard IAA (Sigma-Aldrich, USA) were resuspended in mass-spectrometry (MS) grade methanol (Thomas Baker, Mumbai) and were spotted onto the target plate using 20 mg/ml of 5-Dihydroxybenzoic acid (DHB) (Bruker Daltonik GmbH, Bremen, Germany) matrix in 70% acetonitrile and 0.1% TFA (Loba Chemie, Mumbai). Subsequently, the samples were analyzed using a Bruker ultrafleXtreme MALDI-TOF/TOF mass spectrometer equipped with smartbeam-II laser (Bruker Daltonics, Billerica, MA, USA) operated in positive mode in the mass range of 100–1,700 Da. Spectral acquisition and analysis were performed using FlexControl 3.4 and FlexAnalysis 3.4 software (Bruker Daltonics, Billerica, MA, USA) respectively.

To confirm the identity of the auxin molecule, the fraction isolated from WTG protein was analyzed using high-resolution mass spectrometry (HRMS). To this end, the lyophilized sample was resuspended in acetonitrile and was analyzed on the Brüker micrOTOF™-Q II mass spectrometer.

### Data deposition

The atomic coordinates and structure factors have been deposited in the Protein Data Bank, www.rcsb.org (PDB ID: 5WXU).

## Electronic supplementary material

SUPPLEMENTARY INFO
