# Supplementary material for: A novel function for globulin in sequestering plant hormone: Crystal structure of Wrightia tinctoria 11S globulin in complex with auxin
Source: Sci Rep. 2017 Jul 5;7:4705. doi: 10.1038/s41598-017-04518-7 (PMC5498579; doi:10.1038/s41598-017-04518-7)
Supplement: Supplementary file 1 — SUPPLEMENTARY INFO [file 41598_2017_4518_MOESM1_ESM.pdf]

**A novel function for globulin in sequestering plant hormone: Crystal structure of *Wrightia tinctoria* 11S globulin in complex with auxin**

**Authors:** Pramod Kumar[a], Pooja Kesari[a], Sonali Dhindwal[a], Ashish K. Choudhary[b], Madhusudhanarao Katiki[a], Neetu[a] , Aparna Verma[a], Kiran Ambatipudi[a], Shailly Tomar[a], Ashwani Kumar Sharma[a], Girish Mishra[b] and Pravindra Kumar[a,*]

[a]Department of Biotechnology, Indian Institute of Technology Roorkee, Roorkee, Uttarakhand-247667, India;

[b]Department of Botany, University of Delhi, Delhi-110007, India.

* To whom correspondence should be addressed: Dr. Pravindra Kumar, Department of Biotechnology, Indian Institute of Technology Roorkee, India – 247667, Telephone: +91-1332-286286, E-mail: pravshai@gmail.com

Supplementary Information

**Supplementary Figure S1**

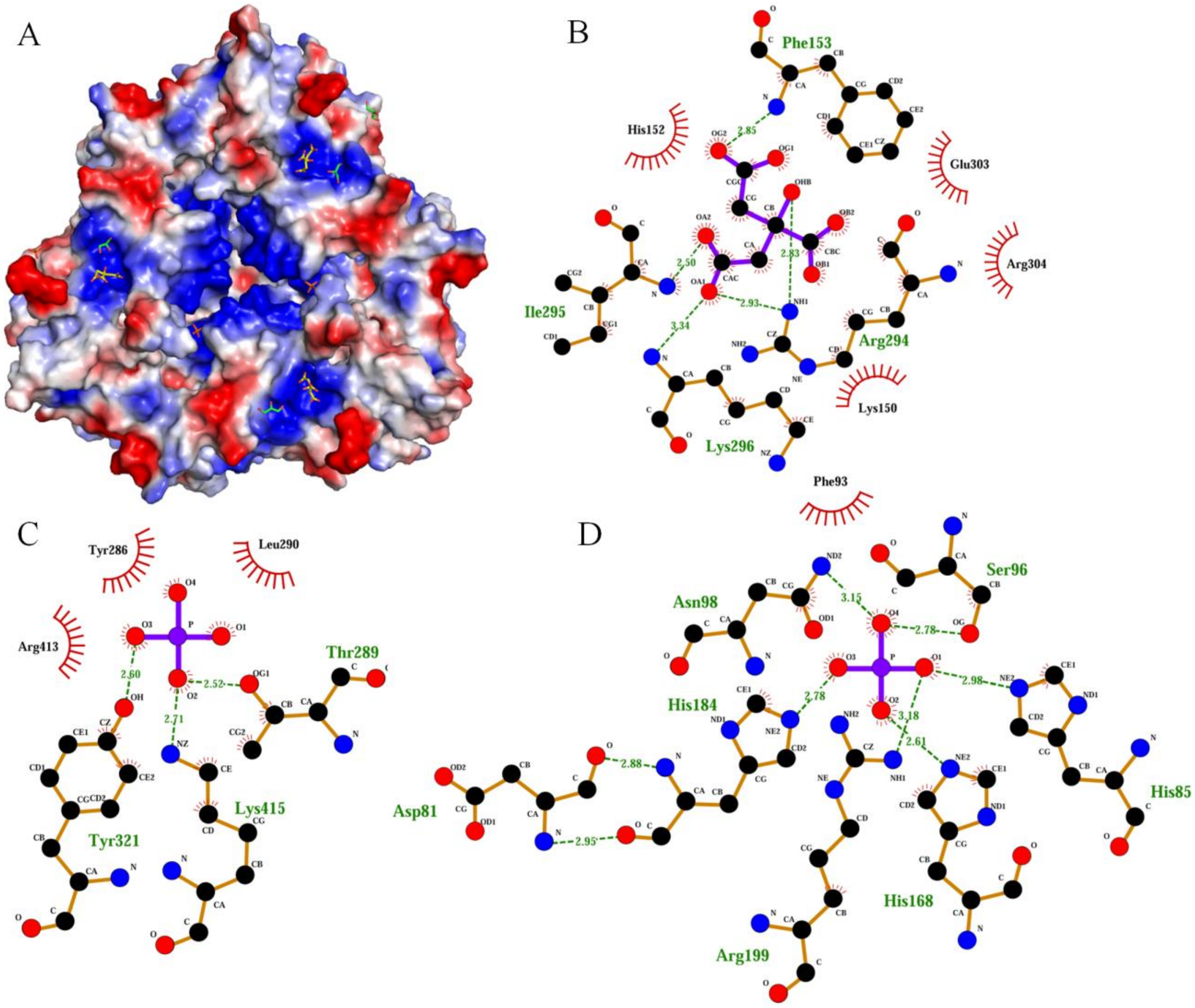


**Figure S1**.The phosphate and citrate ligands bound to IE surface.

**A.** Electrostatic IE surface of the ABC trimer showing the binding site of phosphate (pink), glycerol (green) and citrate (yellow), shown in sticks. **B.** LIGPLOT diagram listing interactions of citrate anion and WTG. **C.** LIGPLOT diagram listing interactions between phosphate and WTG monomer. **D.** LIGPLOT diagram listing interactions between phosphate and adjacent monomers of WTG.

**Supplementary Figure S2**

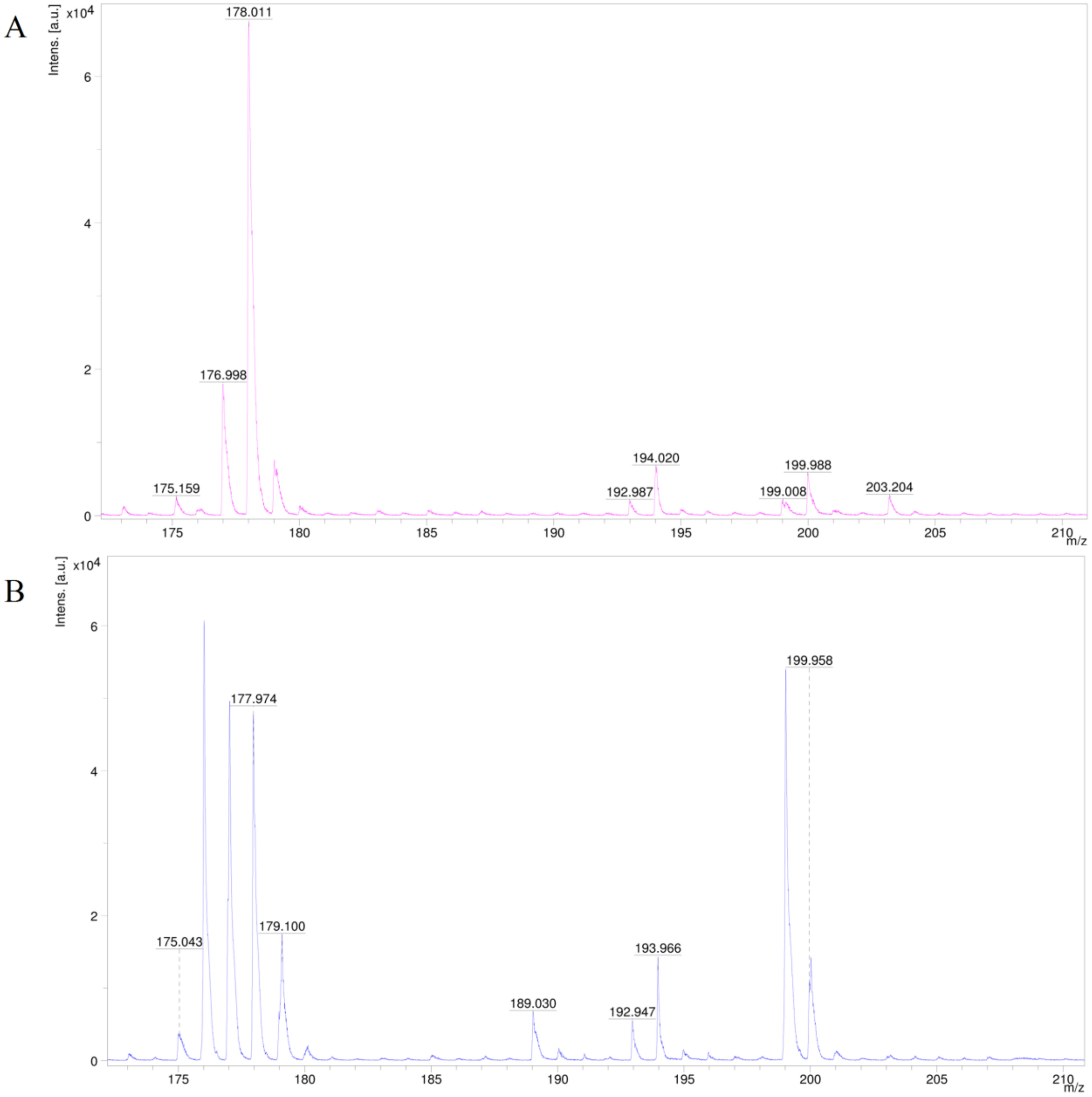


**Figure S2** MALDI-MS spectra profile of IAA obtained from denatured WTG sample (A) and pure Indole-3-acetic acid (IAA) acquired from Sigma-Aldrich (B). The spectrum showed peaks in the range from 172 to 210 m/z indicating peaks corresponding to the matrix ions.

**Supplementary Figure S3**

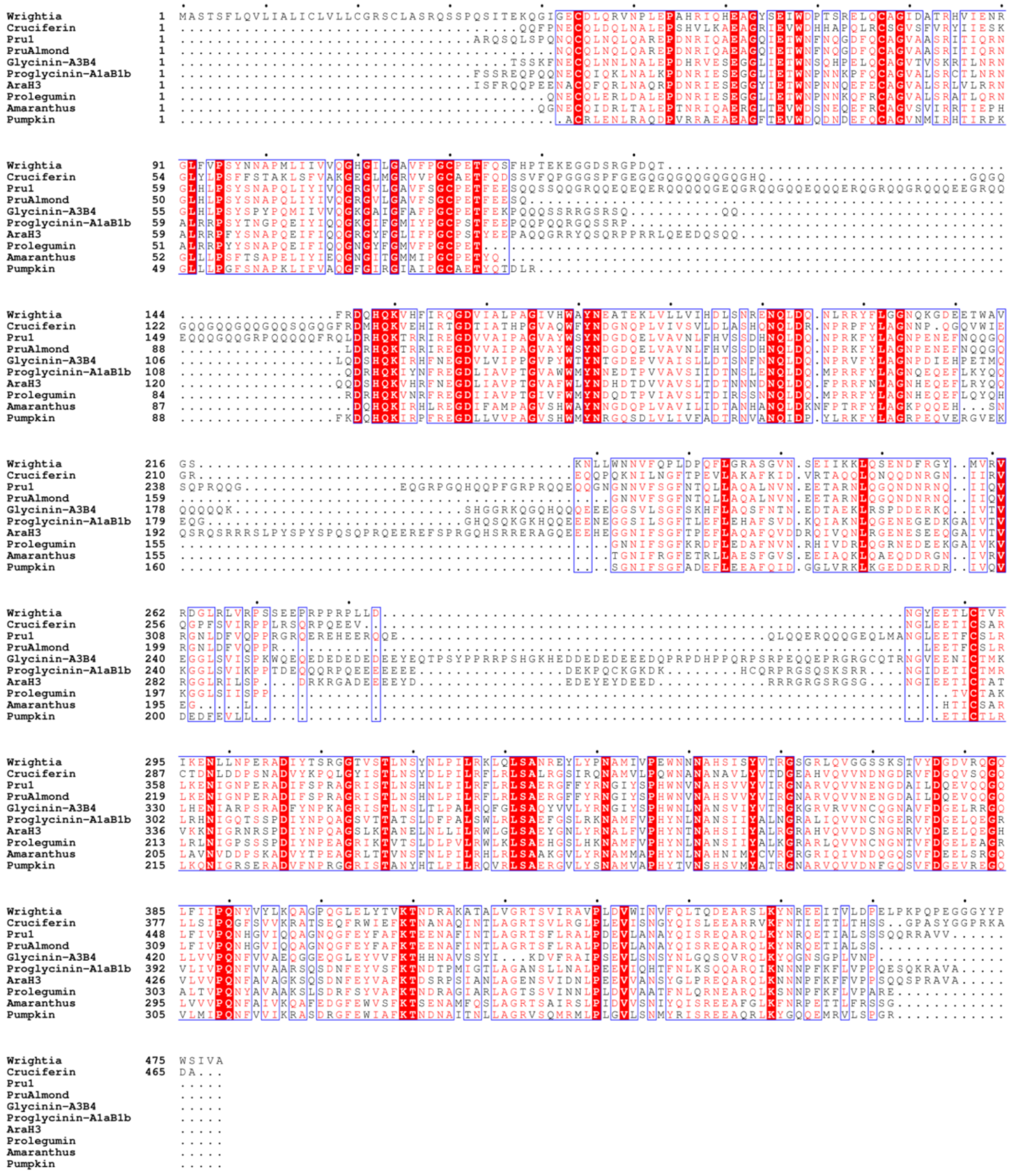

**Figure S3**. Sequence alignment of WTG with other related members of the 11S family. Identical residues are shown in red boxes with white characters; red characters denote identical residues across the column, whereas blue boxes denote similar residues. The sequence alignment was performed using ClustalW1 and the figure was generated using ESPript2. Members of 11S family used to generate sequence alignment are cruciferin (PDB ID: 3kgl), Pru1 prunin (PDB ID: 3fz3), Pru Almond (PDB ID: 3ehk), glycinin-A3B4 (PDB ID: 2d5f), proglycinin-A1aB1b (PDB ID: 1fxz), arachin ARAH3 (PDB ID: 3c3v), prolegumin (PDB ID: 3ksc), Amaranthus (PDB ID: 3qac) and 11S globulin pumpkin (PDB ID: 2evx).

**Supplementary Figure S4**

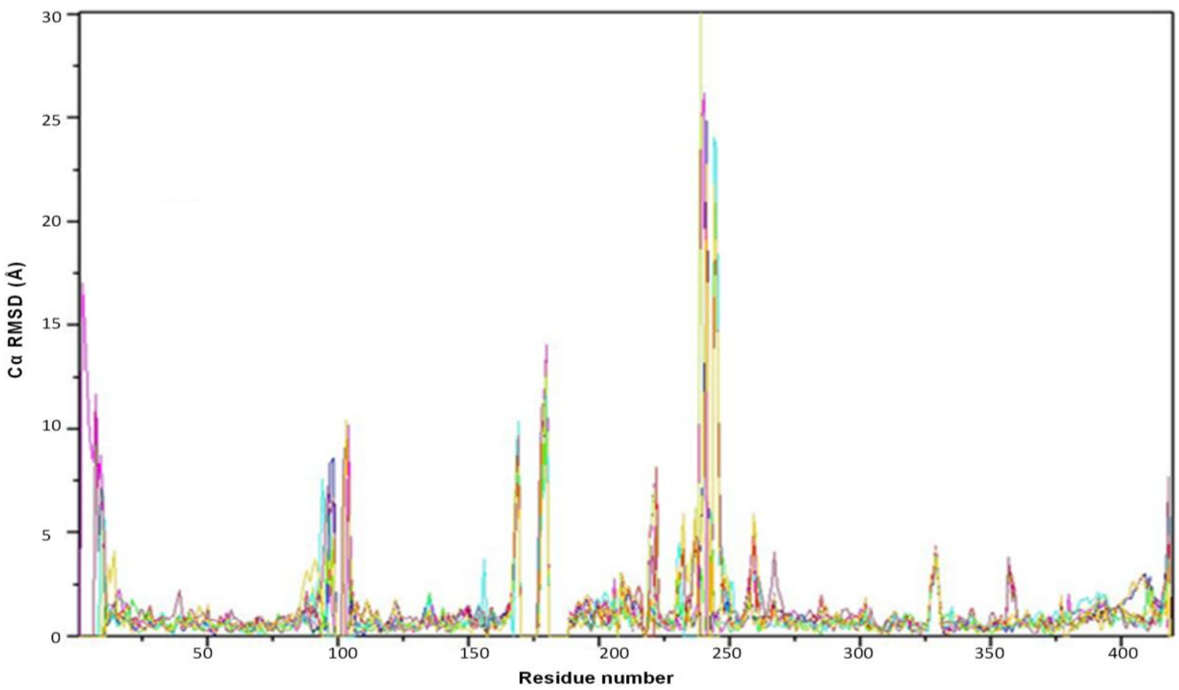


**Figure S4**. $C_\alpha$ RMSD per residue plot of structure of WTG (black) with other 11S globulin structure prunin (PDB ID: 3fz3) (red), Pru Du Amandin (PDB ID: 3ehk) (green), glycinin A3B4 subunit (PDB ID: 2d5f) (blue), arachin ARAH3 (PDB ID: 3c3v) (magenta), glycinin (PDB ID: 1od5) (yellow), prolegumin (PDB ID: 3ksc) (light green), Amaranthus (PDB ID: 3qac) (cyan), 11S proglobulin pumpkin (PDB ID: 2e9q) (golden), procruciferin from *Brassica napus* (PDB ID: 3kgl) (violet), The X-axis corresponds to the residue number whereas Y-axis corresponds to the $C_\alpha$ RMSD. The auxin binding site region corresponds to residue 92-95 and 392-400. The region 92-95 corresponding to residue region Gly119-Glu122 show large RMSD fluctuation whereas region 392-400 corresponding to residue region Arg422- Arg427 show less fluctuation.

**Supplementary Figure S5**

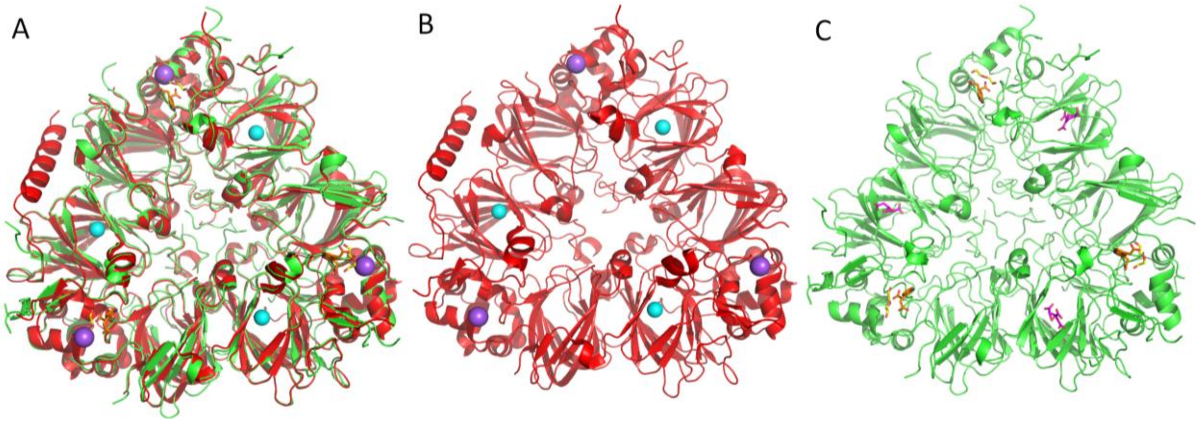


**Figure S5.** Structure comparison of prunin (PDB ID: 3fz3) and WTG.

**(A)** Superimposition of trimer assembly of prunin (red) over WTG (green) shows ligand bound on the IE surface of both the trimers. **(B)** Structure of prunin contains $Ca^{2+}$ (blue sphere) and $Na^{+}$ ion (purple sphere). **(C)** The structure of WTG contains citrate ion (magenta sticks), auxin (orange stick) and PEG ion (yellow stick). The binding site for $Ca^{2+}$ ion in prunin is occupied by citrate ion in WTG. The $Na^{+}$ ion lies in close proximity to the auxin binding site of WTG.
